# Growing Crystals for X-ray Free-Electron Laser Structural Studies of Biomolecules and Their Complexes

**DOI:** 10.3390/ijms242216336

**Published:** 2023-11-15

**Authors:** Christo N. Nanev, Emmanuel Saridakis, Naomi E. Chayen

**Affiliations:** 1 Rostislaw Kaischew Institute of Physical Chemistry, Bulgarian Academy of Sciences, 1113 Sofia, Bulgaria; 2 Institute of Nanoscience and Nanotechnology, National Centre for Scientific Research “Demokritos”, Ag. Paraskevi, 15310 Athens, Greece; 3 Division of Systems Medicine, Department of Metabolism, Digestion and Reproduction, Faculty of Medicine, Imperial College London, London W12 0NN, UK; n.chayen@imperial.ac.uk

**Keywords:** macromolecular crystallization, crystallization theory, microcrystals, X-ray crystallography, X-ray free-electron laser, serial femtosecond crystallography

## Abstract

Currently, X-ray crystallography, which typically uses synchrotron sources, remains the dominant method for structural determination of proteins and other biomolecules. However, small protein crystals do not provide sufficiently high-resolution diffraction patterns and suffer radiation damage; therefore, conventional X-ray crystallography needs larger protein crystals. The burgeoning method of serial crystallography using X-ray free-electron lasers (XFELs) avoids these challenges: it affords excellent structural data from weakly diffracting objects, including tiny crystals. An XFEL is implemented by irradiating microjets of suspensions of microcrystals with very intense X-ray beams. However, while the method for creating microcrystalline microjets is well established, little attention is given to the growth of high-quality nano/microcrystals suitable for XFEL experiments. In this study, in order to assist the growth of such crystals, we calculate the mean crystal size and the time needed to grow crystals to the desired size in batch crystallization (the predominant method for preparing the required microcrystalline slurries); this time is reckoned theoretically both for microcrystals and for crystals larger than the upper limit of the Gibbs–Thomson effect. The impact of the omnipresent impurities on the growth of microcrystals is also considered quantitatively. Experiments, performed with the model protein lysozyme, support the theoretical predictions.

## 1. Introduction

Although conventional X-ray crystallography remains the dominant method for structural determination of proteins and other biomolecules, two challenges are facing the conventional approach: (1) the notorious difficulty of growing large and well-diffracting protein crystals, and (2) the radiation damage caused by exposure to X-rays. Due to radiation damage, crystals of very small size (<10 μm) are difficult to examine using a synchrotron and, typically, X-ray diffraction has to be performed at cryogenic temperatures.

To overcome these challenges [1], the burgeoning method of time-resolved structural studies of biomacromolecules and their complexes using free-electron lasers (FELs) provides an attractive alternative. Serial femtosecond crystallography (SFX) uses extremely intense X-ray pulse irradiation of small crystals [2]. Solem [3] already demonstrated that, at sufficiently high X-ray intensity, an image of diffraction-limited resolution can be captured before the specimen is obliterated. This ”diffraction-before-destruction” approach [4,5] is used by SFX; in a series of femtosecond X-ray pulses, FELs deliver beam intensities of more than ten orders of magnitude greater than synchrotron light sources [6]. Therefore, SFX allows for collection of high-resolution data at room temperature [7]. The short pulse durations of SFX also remove the effect of atomic motion. Importantly, X-ray free-electron lasers (XFELs) not only provide 3D structures of proteins and macromolecular complexes, but also permit time-resolved studies of protein–protein interactions. Furthermore, XFELs can overcome the obstacles to RNA structural determination faced by conventional X-ray crystallography, NMR, and cryoelectron microscopy [8].

XFEL is implemented by irradiating microjets of microcrystalline suspensions with extremely intense femtosecond X-ray pulses. Because the crystals are randomly oriented, the individual snapshot diffraction patterns resemble 3D powder diffraction patterns. As sample delivery for serial crystallography at free-electron lasers is of prime importance, the issue has been considered in detail [9,10]. Basic set up and procedures for microfluidic mix-and-liquid-injection used for time-resolved structure determination of macromolecular conformations and ligand-bound intermediates have also been reported [11]. Most recently, mix-and-inject serial crystallography was used for the direct observation of structural changes associated with ongoing enzymatic reactions [12].

As for conventional X-ray crystallography, the success of experiments with XFELs depends on the ability to grow high-quality crystals, in this case nano- or microcrystals that are delivered to the FEL beam in a liquid stream of their mother liquor [13]. However, much less attention has been given to growing these microcrystals. Kupitz et al. [13] noted that “While methods to grow large single crystals for standard X-ray crystallography have been extensively explored, methods for growth of high-quality nano/microcrystals suitable for SFX experiments are highly desired, yet largely unexplored”. Kupitz et al. [13] also emphasized that, because the surface-to-volume ratio is much higher for nanocrystals than for larger, micrometer-sized crystals, a net transfer of protein from the small to the larger crystals can occur. While the process (known as Ostwald ripening) is very slow when the crystal suspension is stored with minimal vibration under diffusion and/or convection-controlled conditions, vibration and shaking cannot be avoided during transport by air and on land, leading to changes in the size distribution; in the worst-case scenario, most of the small crystals completely dissolve and the remaining crystals are too large for XFEL data collection [13].

Batch crystallization is predominantly used for preparing the microcrystalline liquid slurries needed for XFEL crystallography [14,15,16]. The mother liquor is then filtered to retain only the suitable crystals [2]. However, to reduce consumption of valuable protein (a serious problem for XFEL crystallography), it is preferable to directly grow crystals with the desired sizes, instead of separating them by filtration, which leads to wasting the larger and smaller crystals as well as imposing undesired shear stress and potential mechanical damage on the selected ones [15]. Furthermore, in order to avoid blockage of the microjet injectors, the crystals used must be highly homogeneous in size. To this end, the mean size of the grown crystals, and the time required for growing crystals to the desired size are considered.

The purpose of the present work is thus to provide a theoretical analysis and some specific directions for growing crystals suitable for XFELs. This is a crucial prerequisite in the process of structural determination by XFEL crystallography, which has been seriously overlooked, as most work in the field of biological crystallogenesis has so far exclusively focused on the production of a small yield of large crystals. Furthermore, impurity inclusion in the grown crystals is considered from a novel point of view, again in relation to the kind of crystals required for XFELs.

## 2. Results

### 2.1. Quantitative Relationship between Crystal Number Density and Mean Crystal Size

The relationship between number density and mean size of crystals has already been discussed elsewhere [17,18]. These calculations concerned the case where crystallization proceeds in solutions in which the amount of dissolved substance is preset, and only a defined part of this substance is incorporated in different numbers and sizes of growing crystals during crystallization. Since the more numerous the crystals, the less the available solute (which is necessary for their growth to large sizes), the numbers and sizes of the growing crystals must be inversely related.

In batch crystallization, provided that the total crystal number density *N*, i.e., the number of crystals in 1 cm^3^ of solution, established during the nucleation stage remains constant during the subsequent crystal growth, the quantitative relationship between *N* and the mean crystal size *l* (cm) can be calculated easily by assuming a cubic crystal shape [17,18]. Under such conditions, at any time point during the crystal growth, the cumulative volume of all growing crystals totals *Nl*^3^, where *l* is the *approximate* edge length of a cubic crystal reached at that point of growth: To calculate *l*, we consider the mass of the uncrystallized solute. While the initial crystallizable mass *m*_o_ (g) decreases gradually during the growth of the crystals, an uncrystallized mass *m*_t_ (g) remains in the solution at any time point *t* during the growth process. Thus, the difference (*m*_o_ − *m*_t_) is the mass of solute that is consumed to grow *N* crystals to the mean size *l.* To convert the cumulative crystalline volume *Nl*^3^ into mass, we divide *Nl*^3^ by the specific volume *υ* (cm^3^/g):

(1) $$l=[\upsilon (m_{o}-m_{t})/N{]}^{\frac{1}{3}}$$

However, solute concentration diminishes constantly during growth of the crystals, and a point at which the solute concentration approaches the solubility *c*_e_ (when no crystal growth is possible) is eventually reached; the maximum achievable mean crystal size *λ* is thus:

(2) $$\lambda =[\upsilon (m_{o}-m_{m})/N{]}^{\frac{1}{3}}$$

where *m*_m_ is the mass that (approximately) corresponds to solubility *c*_e_.

Finally, dividing Equation (1) by Equation (2), we obtain *l*:

(3) $$l=\lambda \{\left[\frac{m_{o}-m_{t}}{m_{o}-m_{m}}\right]{\}}^{\frac{1}{3}}=\lambda \{\left[\frac{c_{o}-c_{t}}{c_{o}-c_{e}}\right]{\}}^{\frac{1}{3}}$$

where *c*_t_ corresponds to *m*_t_.

Equations (1) and (2) enable designing crystallization trials aimed at growing crystals suitable for XFELs: It is seen that *l* and *λ* are inversely proportional to *N*^1/3^. This dependence is very weak, and even an approximate *N* value can enable estimation of the desired crystal sizes *l* and the maximum achievable crystal size *λ*. (Due to the stochastic nature of the nucleation process, such an estimate is sufficient.) Knowing *c*_e_ and *υ* of the crystallizing substance, Equation (2) enables estimation of the solute mass *m*_o_, which is needed to obtain the desired theoretical crystal yield *N*λ^3^. If the time required to reach solubility *c*_e_ proves to be unacceptably long, there is the possibility to stop the crystallization process at any desired (mean) crystal size *l*, according to Equation (1). To this end, the time needed for growing crystals to size *l* is calculated as described in the following subsection.

### 2.2. Growing Crystals Suitable for X-ray Free-Electron Laser Studies

The change in crystal size is obtained by differentiating Equation (1). As *m*_o_ is a constant, we obtain:

(4) $$dl=-\frac{\upsilon }{3l^{2}N}dm_{t}$$

Evidently, the change in *l* depends on the change in the uncrystallized mass *m*_t_. The latter diminishes during the growth of the crystal, but the effect of this decrease (i.e., a decrease in the driving force for growth) is somewhat counterblanced by the increase in the crystal surface.

To obtain the growth rate of a crystal of size *l*, Equation (4) is rewritten in the form:

(4.1) $$\frac{dl}{dt}=-\frac{\upsilon }{3l^{2}N}\frac{dm_{t}}{dt}$$

Similarly, differentiating Equation (2), and since *m*_o_ and *m*_m_ are constants, we obtain:

(5) $$d\lambda =0,\;\mathrm{and}\;\frac{d\lambda }{dt}=0$$

This result reflects the statement that no crystal growth is possible after *λ* is reached.

Due to Ostwald ripening, incubation times longer than necessary can result in growing crystals that are too large [13] and block the crystal injectors for XFELs. We therefore consider the growth of the critical nucleus (of edge length l*) of a cubic crystal, until it reaches a desired crystal size *l*_1_. The process of crystal growth can be conceptually divided in two consecutive stages: the first covers the growth of the crystal from size l* until the maximum crystal size *L*_max_ for which the Gibbs–Thomson law is valid. (According to the Gibbs–Thomson law, small clusters of molecules, including crystals, are in equilibrium with their mother phase at a higher supersaturation than larger crystals). The second stage is the growth of that crystal beyond *L*_max_, to its final size. The time for growth from l* to *L*_max_ is denoted *τ*_1_, while the final size *l*_1_ is reached after additional growth time *τ*_2_. Thus, time (*τ*_1_ + *τ*_2_) is needed for growing crystals larger than those corresponding to the upper limit of the Gibbs–Thomson effect.

Stage 1. Beginning of crystal growth, from a nucleus of size l* until *L*_max_.

Under conditions where crystal growth proceeds purely by diffusion of solute to the crystal surface, the time *τ*_1_ can be calculated using the equation for the rate of crystal growth [19]:

(6) $$\frac{dm}{dt}=\left(\frac{SD}{\delta_{N}}\right)\left(c_{t}-c_{e}\right)$$

where *S* is the crystal total surface area, *D* (cm^2^/s) the diffusion coefficient of the solute, and *δ*_N_ [cm] the thickness of the Nernst diffusion layer [20]; *c*_t_ is the actual solute concentration at the surface of the growing crystal, and *c*_e_ is the equilibrium concentration with respect to an “infinitely” large crystal.

To calculate the rate of crystal growth according to Equation (6), we use the following approximation for the difference ∆μ between the chemical potentials of the two phases (crystal and solution) [21], which holds true for small and medium supersaturations during solution crystal growth (note that while high supersaturation is required for crystal nucleation, it is advisable to significantly reduce the supersaturation for growth of higher quality crystals [22]):

(7) $$∆\mu \approx \;k_{B}T(\frac{{c_{t}-c}_{e}}{c_{e}})$$

where *k*_B_ is the Boltzmann constant and *T* the absolute temperature.

According to the classical crystal nucleation theory, the edge length l* of the cubic crystal nucleus, i.e., the crystal which remains in equilibrium with the solution, is $l^{*}=\frac{4\Omega \gamma }{∆\mu }$, where *Ω* is the volume of the crystal building block, and *γ* the specific energy of the interface between crystal nucleus and its surroundings. Thus, $l^{*}=\frac{4\Omega \gamma c_{e}}{k_{B}T({c_{t}-c}_{e})}$ and

(8) $$c_{t}-c_{e}=\frac{{4\Omega \gamma c}_{e}}{k_{B}Tl^{*}}$$

As the crystal growth rate changes constantly (see Equation (4)), in order to calculate the time *τ*_1_ for isothermal crystal growth from a crystal nucleus of negligible size to a crystal of size *l*, we use the average value ${\left(\frac{dm}{dt}\right)}_{avg}$:

(9) $${\left(\frac{dm}{dt}\right)}_{avg}=(\frac{1}{S})\int_{0}^{S}\left(\frac{SD}{\delta_{N}}\right)\left(c_{t}-c_{e}\right)dS$$

For nano- and microcrystals that obey the Gibbs–Thomson law, l* interrelates *S* and $\left(c_{t}-c_{e}\right)$ in Equation (9). Importantly, while the growth of all crystals leads to a gradual decrease in concentration throughout the crystallizing system, the growth of a crystal of edge *l* leads to a decrease in the concentration merely around the said crystal. This assumption is valid for the case when the crystals are sufficiently far from each other. (Although the situation when hundreds of crystals grow simultaneously very close together differs, the result of the following calculation is informative; see below). Thus, with the increase in *S* = Kl*^2^, where *K* is the number of faces, the solute concentration around the growing crystal simultaneously decreases, i.e., $\left(c_{t}-c_{e}\right)$ diminishes. For cubic crystals *K* = 6, and substituting $S=6{l^{*}}^{2}$ and $c_{t}-c_{e}=\frac{{4\Omega \gamma c}_{e}}{k_{B}Tl^{*}}$, we obtain Equation (10) from Equations (8) and (9):

(10) $${\left(\frac{dm}{dt}\right)}_{avg}=\frac{16\Omega \gamma c_{e}Dl^{*}}{{k_{B}T\delta }_{N}}$$

Incorporating mass *m* for time *τ*_1_, the crystal of size l* grows to size *L*_max_. As *m* is obtained by multiplying ${\left(\frac{dm}{dt}\right)}_{avg}$ by *τ*_1_, if we multiply both sides of Equation (10) by *τ*_1_ and replace l* with *L*_max_, we obtain:

(11) $$L_{max}=\frac{m\delta_{N}k_{B}T}{16\Omega \gamma c_{e}D\tau_{1}}$$

or

(12) $$\tau_{1}=\frac{m\delta_{N}k_{B}T}{16\Omega \gamma c_{e}DL_{max}}$$

As may be expected intuitively, *L*_max_ depends merely on *m*/*τ*_1_—all other factors are constants. 

As $m={L_{max}}^{3}\rho$, where *ρ* (g/cm^3^) is the density of the crystal, we can also write:

(12.1) $$\tau_{1}=\frac{L_{max}^{2}{\rho \delta }_{N}k_{B}T}{16D\Omega \gamma_{c}c_{e}}$$

Evidently, since the crystal grows (and dissolves) by attachment (or detachment) of its building blocks at its surface, *τ*_1_ depends proportionally on the surface area.

Importantly, Equation (11) has been derived for crystals that are far apart from each other; for closely spaced crystals, $\left(c_{t}-c_{e}\right)$ drops more rapidly and the amount of *m* incorporated into the crystal during the same time *τ*_1_ is smaller. Very roughly, *m*/*τ*_1_ decreases in proportion to the number of surrounding crystals, but it also depends on their relative distances. Thus, the relation *m*/*τ*_1_ is case specific, and can hardly be determined with any precision.

Stage 2: Growth of crystals larger than *L*_max_.

Of course, the crystal growth rate changes constantly also during the growth of crystals larger than *L*_max_. Therefore, to calculate the additional growth time *τ*_2_ for the crystal to grow to size *l*_1_ > *L*_max_, we use again the average growth rate ${\left(\frac{dm}{dt}\right)}_{avg}$ expressed by Equation (9). Knowledge of the quantitative relationship between *S* and $c_{t}-c_{e}$ is again needed. This relationship is provided in Equation (1), rewritten in the form:

(1.1) $$m_{t}=m_{o}-\frac{Nl^{3}}{\nu \upsilon }$$

Subtracting from both sides of this equation the mass *m*_m_ (which is the mass of solute at solubility *c*_e_), we write:

(1.2) $$m_{t}-m_{m}=m_{o}-\frac{Nl^{3}}{\upsilon }-m_{m}$$

and dividing ${(m}_{t}-m_{m}$) by the volume *V* of the crystallizing droplet, we obtain the supersaturation $\left(c_{t}-c_{e}\right)$ that drives the growth of a crystal of size *l*:

(1.3) $$c_{t}-c_{e}=c_{o}-\frac{Nl^{3}}{\upsilon V}-c_{e}\;$$

Again, with the increase in the crystal surface *S* = *6l*^2^ (for cubic crystals), the solute concentration around the growing crystal simultaneously decreases, i.e., $\left(c_{t}-c_{e}\right)$ diminishes. Substituting $S={6l}^{2}$ in Equation (9), we obtain the average crystal growth rate ${\left(\frac{dm}{dt}\right)}_{avg}$ for crystals of average size *l*—starting from negligible size, i.e., from *l* ≈ 0, right up to the attainment of $c_{e}$:

(14) $${\left(\frac{dm}{dt}\right)}_{avg}=\frac{12D}{\delta_{N}l^{2}}\int_{0}^{l}l^{3}\left(c_{o}-\frac{Nl^{3}}{\upsilon V}-c_{e}\right)dl$$

As in general the mechanism that controls the growth of crystals (i.e., diffusion, kinetic, or mixed control) is unknown, the importance of Equation (14) is that it determines a crystal growth rate that does not depend on any specific crystal growth mechanism and is in this sense universal.

As *D*, *δ*_N_, *N*, υ, *V*, *c*_o_, and *c*_e_ are considered constant, performing definite integration, the solution of Equation (14) is:

(15) $${\left(\frac{dm}{dt}\right)}_{avg}=\frac{3Dl^{2}}{\delta_{N}}(c_{o}-c_{e}-\frac{4Nl^{3}}{7\upsilon V})$$

Equation (15) again accounts for the fact that crystals grow at their surfaces; this is reflected by $l^{2}$, while the term $\frac{4Nl^{3}}{7\upsilon V}$ (which accounts for the total volume of the grown crystals) reflects the appreciable decrease in $c_{o}$ due to the growth of all crystals.

Now, multiplying the average value of the crystal growth rate expressed by Equation (15) by the time from zero to *τ*, during which the crystal grows from negligible size to size *l* (by adding mass *m*), we obtain:

(16) $$\tau =\frac{m\delta_{N}}{3Dl^{2}(c_{o}-c_{e}-\frac{4Nl^{3}}{7\upsilon V})}$$

This equation enables calculation of the time $\tau_{2}=\tau -\tau_{1}$ that is needed to grow crystals to any desired size *l*_1_ > *L*_max_, i.e., above the upper limit of the Gibbs–Thomson effect ($\tau_{1}$ being the time during which the crystal grows to size *L*_max_).

To calculate the increase in the surface of the growing cubic crystal from *L*_max_ to *l*_1_ for time *τ*_2_, we rewrite Equation (16) as:

(17) $$l^{2}=\frac{m\delta_{N}}{3D\tau (c_{o}-c_{e}-\frac{4Nl^{3}}{7\upsilon V})}$$

So, we have:

(18) $$6\left(l_{1}^{2\;}-L_{max}^{2}\right)=\frac{2m\delta_{N}}{D}[\frac{1}{\tau \left(c_{o}-c_{e}-\frac{4Nl_{1}^{3\;}}{7\upsilon V}\right)}-\frac{1}{\tau_{1}\left(c_{o}-c_{e}-\frac{4Nl^{3}}{7\upsilon V}\right)}]$$

For simplicity, the calculation is made here for cubic crystals, but due to the 2/3 surface-to-volume scaling with *l* for all polyhedral crystals, Equations (11), (12), and (16) must also be valid for other crystal shapes—substituting, of course, the corresponding numerical coefficients *K*.

The grown crystals are never equally sized. Their size distribution is considered in Appendix A.

### 2.3. Impurity Inclusion in the Grown Protein Crystals

All considerations conducted so far concern idealized crystallizing systems—those in which there are no impurities. However, especially with proteins, this is never the case; impurities are always present in any protein solution. In the following, we consider the effect of impurity inclusion in the growing protein crystals. For such crystals, impurities are predominantly of biological origin, and are present in every protein solution (including the most highly purified ones). Such impurities typically are remnants of source biomaterial, other protein species, noncrystalline protein aggregates, or traces of nonprotein biomacromolecular impurities.

It is believed that the frequently observed premature termination of protein crystal growth is due to the buildup of impurities, leading to poisoning of the growing crystal faces. This phenomenon means that the maximum achievable mean crystal size λ is reached before solubility is attained (it can therefore formally be accounted for by placing in Equation (2) a larger mass than the one that corresponds to the solubility). Qi and Wakayama [23] suggested that convective flows (so-called plumes) that bring additional impurities to the surface (which are added to those brought by diffusion itself), can be the prime reason for the “crystal growth cessation” phenomenon. However, the easier growth of microcrystalline showers, which are observed in initial screening setups, suggests that small crystals are less prone to crystal surface poisoning than bigger ones. In other words, the impurities can be an obstacle to growing large protein crystals (that are needed for classical X-ray crystallography), but hardly prevent the growth of microcrystals for XFEL crystallography.

The premise for our quantitative consideration of premature protein crystal growth termination is the purely diffusion-controlled growth of protein crystals in microgravity conditions (where typically, more perfect protein crystals are grown). It is suggested that the quiescent crystal growth under such conditions leads to the occurrence of “self-purifying” zones [24]. Such zones arise due to the slow diffusion supply of impurities and, assisted by rejection of impurities, their presence contributes to a more regular attachment of crystal-building blocks, which, in turn, yields crystals of higher quality. The self-purifying zones appear because at the initial stage of growth, the newly created crystal is enriched with impurities that are present in the mother liquor. Indeed, this impurity enrichment within the crystal occurs at the expense of the surrounding solution, and if the latter is stagnant, a zone depleted of impurities appears around the growing crystal; as crystallization proceeds, the solution surrounding the growing crystal becomes increasingly pure [24].

It is logical to assume that under terrestrial conditions also, such self-purifying zones may arise initially around the growing nanocrystals but are destroyed later—due to the stirring effect of the arising convective plumes [25]. Therefore, it is of prime interest to establish the crystal size at which convective plumes start to appear over the growing protein crystals. This will show to what crystal size the Chernov “self-purification zone” around the growing crystals is preserved, i.e., until which point, according to our working hypothesis, impurities and solution agitation do not play a significant role in the crystal growth.

The mass transfer rate with convection is best characterized by the Sherwood number Sh (see ref. [26] (p. 168)). Recall that Sh is a dimensionless concentration gradient at the crystal surface, representing the ratio of the convective mass transfer to the rate of diffusive mass transport toward the microcrystal:

(19) $$Sh=\frac{h}{D/L}$$

where *h* is the convective mass transfer film coefficient (cm/s) and *L* a characteristic length (cm).

For Sh, Wilcox uses Equation (20), see ref. [26] (p. 180):

(20) Sh=2+Sc1/3Re1/2

where 2 is the value of Sh for steady-state mass transfer to a sphere in the absence of convection; Sc is the dimensionless Schmidt number and Re the dimensionless Reynolds number.

The Schmidt number Sc defines the ratio of kinematic viscosity *ν* (cm^2^/s) to mass diffusivity *D* (cm^2^/s), i.e., the ratio of momentum diffusivity to molecular diffusivity:

(21) $$Sc=\frac{\nu }{D}$$

while Re is defined as:

(22) $$Re=\frac{uL}{\nu }$$

Re reflects the ratio of momentum forces to viscous forces: *u* (cm/s) is the flow speed, and *L* (cm) is a characteristic linear dimension (in the case under consideration this is the crystal size).

It is of interest to estimate when, i.e., at what rate *u*, the convective flow above protein crystals of dimensions 5–10 μm starts. (These are typical crystal sizes for XFEL [5]). As already mentioned, the mass transfer is purely diffusive when Sh ≈ 2. Therefore, according to Equation (20), the absence of convective flow requires Sc^1/3^Re^1/2^ <<1. For liquids, *ν* is on the order of 0.01 cm^2^s^−1^ (for instance, the kinematic viscosity of water at 20 °C is 1.003 mm^2^s^−1^), and with the diffusion coefficient for lysozyme *D ≈* 1.06 × 10^−6^ cm^2^/s [27], Sc is, according to Equation (21), of the order of 10^4^. Therefore, Sc^1/3^ ≈ 21.5, and for Sc^1/3^Re^1/2^ << 1, Re^1/2^ must be at least 10^−3^.

Finally, because according to Equation (22):

(22.1) $$u=\frac{\nu Re}{L}$$

with Re = 10^−6^, the speed of the convective flow for *L* = 10^−3^ cm must be *u* = 10^−5^ cm/s. This is a creeping flow for which boundary layer flow and plumes above such crystals are hardly expected. In other words, it is reasonable to assume that the supply of impurities to crystals of size equal or smaller than 10 μm is restricted merely to diffusional supply.

Importantly, the (dimensionless) Peclet number Pe gives the ratio of convective mass transfer to diffusive mass transfer:

(23) $$Pe=ScRe=\frac{uL}{D}$$

From Equation (23), for *u* = 10^−5^ cm/s and *L* = 10^−3^ cm, Pe = 0.01, i.e., the convective mass transfer is only 1% of the diffusive mass transfer, and the larger the crystal, the slower the flow that is sufficient for transferring the same convective mass (amounting to 1%). Indeed, a flow rate *u* = 10^−5^ cm/s (i.e., 0.6 μm/min) for *L* = 10^−3^ cm is hardly measurable, but Pusey et al. [25] observed (and measured) growth plumes above larger lysozyme crystals, of 0.3, 0.5, 1.2, and 1.7 mm across the (110)face. Figure 3 in ref. [25] indeed shows that the apex plume velocities increase with the increase in crystal size. This observation favors our hypothesis that, while impurities brought by convective plumes can be an obstacle for growing large protein crystals, these impurities can hardly stop the growth of microcrystals that are needed for XFEL crystallography.

### 2.4. Experimental Results

Unfortunately, the calculations of *τ*_1_ and *τ*_2_ do not provide an exact answer to the question of how long the growth time must be for reaching the desired crystal sizes. Firstly, due to natural convection [25], the solution around sufficiently large crystals can be replenished, leading to faster growth of these crystals. On the other hand, natural convection brings more impurities to the surface of the growing crystals, which delay crystal growth. These are processes that defy accurate theoretical description. Secondly, the nucleation induction time (if appreciable) must be added to *τ*_1_ and *τ*_2_. To evaluate the overall effect of all these factors and to verify some of the theoretical results, we conducted experimental studies with lysozyme, which, because of the availability of accurate solubility data at various conditions and of the ease with which its crystallization can be fine-tuned and controlled, has become the standard model protein for crystallization studies.

The results from the second series of trials (see Materials and Methods) are displayed in Table 1. It was seen that most crystals were of roughly cubic shape and that the trials displayed high, although not perfect, reproducibility.

At 5% NaCl, we obtain at 24 h (growth completed) from 200 to several hundred crystals with sizes 50–100 μm in each dimension. Thus, *N*λ^3^ ranges from 0.25 × 10^−4^ to 2 × 10^−4^ cm^3^. From Equation (2), *N*λ^3^ = υ(m_0_ − m_m_). The initial mass of lysozyme in 2 μL of a 50 mg/mL solution is *m*_o_ = 0.05 × 2 × 10^−3^ = 10^−4^ g. The solubility of lysozyme in a 5% NaCl solution *c*_e_ = 2.16 mg/mL = 2.16 × 10^−3^ g/cm^3^ [28], so at solubility we have *m*_m_ = 2.16 × 10^−3^ × 0.002 = 0.4 × 10^−5^ g in 2 μL. The density of a lysozyme crystal at 1 M NaCl is *ρ* = 1.24 g/cm^3^ [29], so its specific volume *υ* = 0.81 cm^3^/g. Thus, *N*λ^3^ = *υ*(*m*_o_ − *m*_m_) = 0.81 × (0.9 × 10^−4^) = 0.78 × 10^−4^ cm^3^, which is in excellent agreement with the range of values obtained above from counting and measuring the crystals.

At 6% NaCl, we obtain at 24 h (growth completed) several hundred crystals (i.e., 300–1000) with sizes 25–50 μm in each dimension. Thus, *N*λ^3^ ranges from 300 × (2.5 × 10^−3^)^3^ = 4.7 × 10^−6^ cm^3^ to 1000 × (5 × 10^−3^)^3^ = 1.25 × 10^−4^ cm^3^. This is a much wider range than for the crystals grown from 5% NaCl, but, looking at the data in Table 1 in greater detail, we may assume the lower value is an underestimate, whereas the higher value appears closer to the average situation.

In 6% NaCl, the solubility of lysozyme drops to *c*_e_ = 1.5 × 10^−3^ g/cm^3^ [28]. Therefore, *N*λ^3^ = υ(*m*_o_ − *m*_m_) = 0.81 × (0.05 − 1.5 × 10^−3^) × 2 × 10^−3^ = 0.81 × 0.97 × 10^−4^ ≈ 0.79 × 10^−4^ cm^3^, which is virtually the same as for 5% NaCl and is again in excellent agreement with the above range of values from counting and measuring the crystals.

We can then use Equation (12) to estimate *τ*_1_:

$$\tau_{1}=\frac{m\delta_{N}k_{B}T}{16\Omega \gamma c_{e}DL^{*}}$$

where *k*_B_*T* ≈ 4.05 × 10^−14^ erg per molecule (at 20 °C), *γ*_c_ ≈ 1 erg/cm^2^, *D* ≈ 10^−6^ cm^2^s^−1^, and $\delta_{N}$ ≈ 10^−2^ cm [20]. The lysozyme molecule can be described as a prolate ellipsoid of rotation with axes of lengths 9 and 1.8 nm [30]. Dynamic light scattering gives a hydrodynamic diameter of ca. 3.6 nm, so *Ω* ≈ 10^−19^ cm^3^. Replacing all these in Equation (12), we have:

*τ*_1_ = C(m/*L*_max_) = C[(*L*_max_ ^3^*ρ*)/*L*_max_] = C *L*_max_ ^2^*ρ*,

where C subsumes all other parameters in Equation (12) except m and *L*_max_; C ≈ 1.172 × 10^11^ cm.s/g for 5% NaCl and 1.69 × 10^11^ cm.s/g for 6% NaCl.

Assuming the upper limit of the Gibbs–Thomson effect ≈ 1 μm,

*τ*_1_ ≈ 1.172 × 10^11^ × 1.24 × (10^−4^)^2^ = 1447 s = 0.4 h or 24 min for 5% NaCl.

This is in excellent agreement with our experimental data, since only extremely small lysozyme crystals (seen as ”dots”) are visible up to *t* = 0.5 h.

Since *υ* varies very little with supersaturation and if we assume a constant *L*_max_, $\tau_{1}$ would be, counterintuitively, somewhat longer for 6% NaCl (35 min). However, although the rate of material deposition is overall greater at higher supersaturations, see Equation (6), the number density of crystals is also substantially larger, the crystals are thus appreciably closer to each other and therefore, as noted above, the mass incorporated into each growing crystal for a given time is, in fact, smaller. This variability leads to fluctuations in the actual $\tau_{1}$, which are, however, quite small compared with the usual statistical fluctuations expected in macromolecular crystallization. For an eightfold difference in crystal volume, i.e., *L*_max_ = 0.5–1 μm, $\tau_{1}$ would still only range from 9 to 35 min, the kind of variability that would not be surprising, even between identical trials.

Let us now calculate τ and $\tau_{2}$ from our experimental data. From Equation (16):

(16.1) $$\tau =\frac{m\delta_{N}}{3Dl^{2}(c_{o}-c_{e}-\frac{4Nl^{3}}{7\upsilon V})}=\frac{\rho V_{crys}\delta_{N}}{3Dl^{2}(c_{o}-c_{e}-\frac{4Nl^{3}}{7\upsilon V})}$$

where $V_{crys}$ is the volume of a crystal of size l and V the volume of the crystallization drop.

As above, the starting lysozyme concentration is $c_{o}$= 5 × 10^−2^ g/cm^3^. For a 5% NaCl precipitating solution, $c_{e}$ = 2.16 × 10^−3^ g/cm^3^; *N* = 300 crystals and l = 75 μm = 7.5 × 10^−3^ cm (see Table 1). Replacing these experimental figures in Equation (16.1):

$$\tau \;\approx \;9696\;s\;\approx \;161\;\min=2.6\;h\;\mathrm{and}\;\tau_{2}=\tau -\tau_{1}\approx 137\;\min\approx 2.3\;h$$

Thus, for 5% NaCl, the total time for the growth of crystals (ca. 75 μm in each dimension) is (nucleation induction time) + $\tau_{1}+\tau_{2}$ ≈ (nucleation induction time) + 2.6 h. The nucleation induction time is certainly less than 30 min (since visible microcrystals are present at that time), so the total time of growth to the crystals’ final size is approximately 3 h, which is also in good agreement with the experimental results.

At 6% NaCl, $c_{e}$ = 1.5 × 10^−3^ g/cm^3^, and we may take *N* = 600 crystals and l = 5 × 10^−3^ cm (Table 1). In this case,

*τ* ≈ 937 s ≈ 16 min.

Recalling that $\tau_{1}$ is difficult to ascertain but could be as low as 9 min, $\tau_{2}=\tau -\tau_{1}$ ≈ 7 min. Thus, at 6% NaCl and taking into account the nucleation induction time, the total growth time for lysozyme crystals to their final size of 50 μm (i.e., smaller than for 5% NaCl) is <46 min, which is also in reasonable agreement with the experimental results.

Let us now determine if we can work in the opposite direction, i.e., predict experimental parameters required for a given amount and size of crystals.

Assume we require 10,000 crystals of size 15 μm per μL (these are realistic numbers for XFELs).

So,

*N*λ^3^ = 10,000 × (1.5 × 10^−3^)^3^ = 3.375 × 10^−5^ cm^3^ = *υ*(*m*_o_ − *m*_m_)

Therefore, in 1 μL:

(*m*_o_ − *m*_m_) = (3.375 × 10^−5^)/0.81 = 4.17 × 10^−5^ g = 4.17 × 10^−2^ mg

At 7% NaCl (pH 4.5, 20 °C), the solubility of lysozyme *c*_e_ = 1.23 mg/mL [29], so in 1 μL, *m*_m_ = 1.23 × 10^−3^ mg.

So, *m*_o_ = 4.17 × 10^−2^ + 1.23 × 10^−3^ = 4.29 × 10^−2^ mg in 1 μL, corresponding to *c*_o_ ≈ 43 mg/mL.

This result compares well with the experiment, since 7% NaCl in 40–50 mg/mL lysozyme does indeed give thousands of tiny but visible protein crystals. This protein concentration thus gives the desired crystal mass for a given protein solubility.

This is a useful result but does not allow us to find the protein solubility (i.e., the precipitating agent concentration at a given temperature, pH, etc.) at which many small rather than few larger crystals are produced. For example, at the much lower NaCl concentration of 3% (at pH 4.6, 20 °C), *c*_e_ = 7.4 mg/mL [29], so in 1 μL, *m*_m_ = 7.4 × 10^−3^ mg. Then, *m*_o_ = 4.17 × 10^−2^ + 7.4 × 10^−3^ = 4.91 × 10^−2^ mg in 1 μL, corresponding to *c*_o_ ≈ 49 mg/mL, which is very close to the above despite the sixfold difference in protein solubility between the two conditions. This condition indeed gives almost the same crystal mass, but concentrated within 1–2 crystals in our 2 μL drops.

The required solubility/supersaturation for many small crystals (XFEL crystallography) as opposed to few large crystals (conventional protein crystallography), will therefore have to be determined beforehand from previous knowledge or preliminary small-scale experiments to determine the supersaturation optimal for the purpose. The *m*_0_ obtained only gives a limit below which the risk of crystals being too few and/or too small for XFEL is appreciable. For example, in the worked example above, 7% NaCl was chosen as we already knew that lower supersaturations have yielded crystals that were too few and large for XFEL, whereas higher ones have resulted in amorphous precipitation. 

Let us now see whether some kind of prediction can be made relative to the time of growth. Using *c*_o_ = 43 mg/mL, the lysozyme solubility corresponding to 7% NaCl, and the required sizes and numbers of crystals stated above, we obtain:

*τ* = 173 s ≈ 3 min

We therefore predict that, at such high supersaturations, growth to the (very small) final crystal size is very rapid, the timescale of the process being dominated by the nucleation induction time.

For the sake of comparison, let us predict the time of growth of lysozyme crystals suitable for conventional crystallography, i.e., for 10 crystals of 200 μm in our 2 μL drop. The suitable NaCl concentration is now 4%, for which *c*_e_ = 3.2 mg/mL [29]. We obtain:

*τ* ≈ 7120 s ≈ 119 min

As we could expect, this time is much longer than for the crystals suitable for XFEL crystallography. This figure is again a reasonably close match to our preliminary crystallization experiments. In this case, the nucleation induction time is above 30 min but certainly below 90 min, and the total time for growth of ca. 10 crystals per drop of a somewhat smaller average size of ca. 150 μm, was approximately 3.5 h.

Since *N* scales as the crystallization volume, these results can be extrapolated to any volume of crystallization solution, including the much larger volumes required for XFEL crystallography.

**Figure 1:**
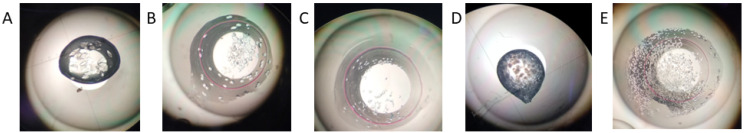
Crystals of hen egg-white lysozyme grown in microbatch at very similar conditions and different incubation times, showing the difficulty of fine-tuning the conditions to the desired result. (**A**) Crystals that are too large for XFEL crystallography but quasi-ideal for conventional crystallography. Grown from 4% NaCl and incubated for 24 h; ca. 150–200 µm in each dimension. (**B**) Crystals that are still too large for XFEL crystallography but can be improved for conventional crystallography. Grown from 5% NaCl and incubated for 24 h; ca. 50–100 µm in each dimension. (**C**) Crystals of approximately adequate size for XFEL crystallography but with too low number density in the solution. Grown from 6% NaCl and incubated for 1.5 h; <40 µm in each dimension. (**D**) Crystals of adequate size for XFEL crystallography growing together in ”hedgehog” clusters, thus making their harvesting very difficult. Grown from 6% NaCl and incubated for 48 h. (**E**) Crystals of adequate size and number density for XFEL crystallography. Grown from 6% NaCl and incubated for 4 h; <25 µm in each dimension.

This is again an interesting result, as it gives an estimate of the time during which a crystallization experiment should be left to incubate. Once again, however, it is only practically useful in designing an experiment if there is some previous knowledge of the concentration of precipitating agent (NaCl in this case) at which the kind of crystals required will be obtained, and of the corresponding nucleation induction time. Figure 1 shows how subtle variations in the concentration of NaCl and incubation time can make the difference between crystals suitable for conventional crystallography, those suitable for XFELs, and those that are not suitable for either method.

## 3. Concluding Remarks

Importantly, crystals of identical symmetry are required for XFEL crystallography. In this respect, it must be noted that protein crystals typically inherit the polymorphic form adopted by the forming nuclei. However, a transition of polymorphic form may also occur at a later crystal growth stage (e.g., see [31]): The well-known Ostwald rule of stages stipulates that the crystallizing phase does not need to be the most stable one thermodynamically; on the contrary, a metastable phase may appear first because crystal nucleus formation requires the surmounting of a lower energy barrier. Afterward, the system may undergo a polymorphic form transition toward another metastable phase or directly to the most stable phase. Therefore, to avoid a change in crystal symmetry, the most stable phase must be determined in preliminary experiments and used for XFEL crystallography.

As already mentioned, large crystals may clog XFEL injectors. A narrow crystal size distribution (CSD) is therefore preferred for XFEL crystallography. The prime cause for crystal polydispersity is the prolonged time during which the crystals nucleate: “Crystal nuclei that form first in the nucleation process have the longest time to grow, and thus, the first-born crystals attain the largest size, while the later nucleated crystals attain smaller and smaller sizes—corresponding to shorter and shorter growing times” [17]. Thus, our recommendation for growing the numerous small crystals needed for XFEL crystallography is to apply the high supersaturation required for nucleation during a short time and then immediately decrease the supersaturation below the supersolubility curve (defined as the curve separating the conditions leading to spontaneous nucleation from the metastable ones): crystal growth occurring in the metastable zone of the Ostwald–Miers phase diagram should lead to production of high-quality crystals suitable for XFEL crystallography.

## 4. Materials and Methods

Two series of crystallization trials were conducted with lysozyme (Sigma-Aldrich, Steinheim, Germany, L6876). In the first series, 100 mg/mL lysozyme in 10 mM sodium acetate pH 4.5 was mixed with various concentrations of sodium chloride in 200 mM sodium acetate, leading to crystallization mixtures of 50 mg/mL lysozyme; 3%, 4%, 5%, 6%, and 8% (*w*/*v*) NaCl (i.e., 30–80 mg/mL NaCl); and 100 mM sodium acetate pH 4.5. Experiments were set up for each condition at two different drop volumes, 2 and 5 μL, and in triplicates for each condition/volume using the microbatch setup under paraffin oil [32] in Douglas Vapor Batch Plates (Douglas Instruments Ltd., East Garston, UK).

Two kinds of solution mixing were tried: (a) salt solution was added into the already dispensed protein solution and mixed in situ, which is what happens in most real protein crystallization situations (referred to as regular mixing), and (b) salt solution was introduced very slowly into a tube containing the protein solution and mixed continuously as it was being added (“pre-mixed”). The second method reduces shock nucleation and premature precipitation. Results of that first set of trials are shown in Table 2.

The 3% NaCl condition remained clear for several days, whereas the 6% under regular mixing and 8% NaCl gave heavy precipitate. These conditions were therefore not pursued further. All other crystallization drops were observed under the stereoscope at *t* = 0, 1.5, 2.5, 4 h, and after complete cessation of growth (*t* = 48 h), and the crystals were counted and measured. However, as the number densities of crystals were still too low compared with the yields needed for XFEL crystallography, a second series of trials was set up.

In the second series, the same lysozyme stock solution was used after having remained refrigerated for ca. 2 weeks. It was known to us from previous experience that such ”aged” lysozyme solutions yield good crystals that, however, tend to nucleate more abundantly. As the 4% NaCl solution of the first series had yielded crystals that were much fewer and larger than what is needed for XFEL crystallography, and the 8% NaCl solution precipitated, we only dispensed 5% and 6% NaCl conditions, with final concentrations of 50 mg/mL lysozyme and 100 mM sodium acetate pH 4.5, as before. This time we only used ”pre-mixed” solutions, which were then centrifuged for 3 min to eliminate the light precipitate that formed upon mixing, which was another problem in the first series of trials. Neither solution displayed measurable loss of protein after centrifugation, within the accuracy of the NanoDrop^TM^ (Thermo Fischer Scientific Inc., Markham ON, Canada) protein concentration measurements that were made. As 2 μL droplets had yielded a higher crystal density than the 5 μL ones in the first set of trials, only 2 μL drops were set (in triplicates) for this series.

## Figures and Tables

**Table 1:** Numbers and sizes of lysozyme crystals for two different precipitating agent concentrations and at different times of incubation after setup; (a)–(c) correspond to each drop of the triplicates set up at each condition, wherever these drops are not identical.

	5% NaCl	6% NaCl
*t* = 0	clear after centrifugation	clear after centrifugation
*t* = 0.5 h	tiny visible crystals	tiny visible crystals
*t* = 1.5 h	(a) 100s of crystals 50 × 50 × 50 μm (b) ca. 200 crystals 75 × 75 × 75 μm (c) ca. 200 crystals 50 × 50 × 50 μm	(a) 100s of crystals, 25 × 25 × 25 μm (b) 100s of crystals (but fewer than a), 25–50 μm in each dimension (c) 100s of crystals (but fewer than a), 25–50 μm in each dimension
*t* = 3.5–4 h	(a) as at *t* = 1 h 30 min (b) ca. 200 crystals 75–100 μm in each dimension (c) as at *t* = 1 h 30 min	(a) as at *t* = 1 h 30 min (b) as at *t* = 1 h 30 min (c) as at *t* = 1 h 30 min
*t* = 45–48 h (growth completed)	(a) 100s of crystals 50 × 50 × 50–75 × 50 × 50 μm (b) ca. 200 crystals 75–100 μm in each dimension + very small ones (<25 μm) (c) as at *t* = 1 h 30 min	(a) as at *t* = 1 h 30 min (b) as at *t* = 1 h 30 min (c) 100s of crystals (but fewer than a), 25–75 μm in each dimension

**Table 2:** Numbers and sizes of lysozyme crystals for various different precipitating agent concentrations and at different times of incubation after setup, in the first series of trials (see Materials and Methods); (a)–(c) correspond to each drop of the 2 μL triplicates and (i)–(iii) to each drop of the 5 μL triplicates set up at each condition.

	3% NaCl	4% NaCl (Regular)	4% NaCl (Pre-Mixed)	5% NaCl (Pre-Mixed)	6% NaCl (Regular)	6% NaCl (Pre-Mixed)	8% NaCl
*t* = 0	All clear for at least 10 days	Shock nucleation, then slowly clarified	clear	Very light precipitate, then slowly clarified	Heavy precipitate everywhere—no crystals	Light precipitate	Heavy precipitate everywhere—no crystals
*t* = 1.5 h		(a) 18 xtals, 50 × 25 × 25–75 × 50 × 50 μm (b) 30 xtals, 75 × 30 × 30–75 × 75 × ? μm (c) 16 xtals, 75 × 25 × 25–75 × 50 × 50 μm (i) 32 xtals, 50 × 25 × 25–75 × 75 × ? μm (ii) 58 xtals, 50 × 30 × 30–75 × 75 × ? μm (iii) 45 xtals, 50 × 30 × 30–75 × 50 × 50 μm	(a) 12 xtals, 30 × 20 × 20–50 × 40 × 40 μm (b) 13 xtals, 25 × 25 × 25–35 × 35 × 35 μm (c) 5 xtals, 50 × 25 × 25–50 × 40 × 40 μm (i) 10 xtals, ca. 30 × 20 × 20 μm (ii) 16 xtals, 50 × 25 × 25–75 × 50 × 50 μm (iii) 19 xtals, 25 × 25 × 25–50 × 50 × 50 μm	(a) 26 xtals, 50 × 25 × 25–50 × 50 × 50 μm (b) 34 xtals, 50 × 25 × 25–50 × 50 × 50 μm (c) 43 xtals, 30 × 15 × 15–50 × 50 × 50 μm (i) 90 xtals, 50 × 25 × 25–50 × 50 × 50 μm (ii) 70 xtals, 50 × 25 × 25–50 × 50 × 50 μm (iii) 67 xtals, 50 × 25 × 25–75 × 50 × 50 μm		(a) 38 xtals, 25–50 μm in each dimension (b) 43 xtals, same (c) 38 xtals, same (i) ca. 65 xtals, same (ii) ca. 70 xtals, same (iii) ca. 70 xtals, same (plus light precipitate)	
*t* = 2.5 h		(a) 24 xtals, 75 × 50 × 50–125 × 75 × ? μm (b) 35 xtals, 125 × 50 × 50–125 × 125 × ? μm (c) 18 xtals, 75 × 50 × 50–125 × 125 × ? μm (i) 49 xtals, 125 × 50 × 50–175 × 125 × 125 μm (ii) 66 xtals, 50 × 50 × ?–125 × 125 × ? μm (iii) 65 xtals, 75 × 50 × 50–125 × 75 × 75 μm					
*t* = 3.5–4 h			(a) 13 xtals, 100 × 100 × 50–180 × 100 × 100 μm (b) 14 xtals, 125 × 125 × 125–180 × 125 × 125 μm, but also some smaller ones (125 × 50 × 50 μm) (c) 9 xtals, 125 × 50 × 50–150 × 150 × 125 μm (i) 15 xtals, 125 × 100 × 100–125 × 125 × 125 μm (ii) 22 xtals, 100 × 100 × 100–160 × 160 × 160 μm (iii) 21 xtals, 125 × 100 × 100–180 × 180 × ? μm	(a) 30 xtals, 150 × 100 × 100–150 × 150 × 150 μm (b) 37 xtals, 150 × 125 × 100–200 × 200 × 200 μm (c) 49 xtals, 125 × 100 × 60–175 × 125 × 125 μm (i) ca. 100 xtals, 125 × 75 × 75–180 × 180 × ? μm (ii) ca. 80 xtals, 180 × 100 × 100–200 × 150 × 150 μm (iii) ca. 85 xtals, 180 × 100 × 100–180 × 180 × 180 μm			
*t* = 45–48 h (growth completed)		(a) 25 xtals, with 2 distinct morphologies: 180 × 180 × 180 and 180 × 125 × 50 μm (b) 31 xtals, as in A1 but there are 2 much smaller ones (ca. 75 × 75 × 50 μm) (c) 17 xtals, 200 × 125 × 125–200 × 200 × 180 μm (i) ca. 55 xtals, 200 × 200 × ? μm (ii) ca. 60 xtals, 200 × 125 × ?–200 × 200 × ? μm (iii) ca. 60 xtals, ca. 180 × 180 × 125 μm	(a) 12 xtals, 225 × 225 × ?–225 × 225 × 225 μm (b) 14 xtals, 250 × 180 × 180–200 × 200 × ? μm, but also some smaller ones (180 × 180 × 125 μm) (c) 9 xtals, 250 × 180 × ?–200 × 200 × ? μm (i) 17 xtals, 125 × 100 × 100–125 × 125 × 125 μm (ii) 22 xtals, 100 × 100 × 100–160 × 160 × 160 μm (iii) 21 xtals, 125 × 100 × 100–180 × 180 × ? μm	(a) 34 xtals, with 2 distinct morphologies: 200 × 180 × 180–180 × 180 × 100 μm (b) 40 xtals, 125 × 125 × 125–180 × 180 × ? μm (c) 65 xtals, 100 × 100 × 100–150 × 125 × 125 μm (i) ca. 90 xtals, 180 × 180 × 180 μm (mostly) (ii) ca. 85 xtals, 125 × 125 × 125–225 × 180 × 180 μm (iii) ca. 75 xtals, 200 × 125 × 125–200 × 200 × 150 μm		All drops had both single crystals and “hedgehog” clusters over them. That made the single crystals impossible to count. Only dimensions could be measured: (a) 75 × 50 × 50–100 × 75 × 75 μm (b) 75 × 75 × ? 125 × 50 × 50 μm (c) 125 × 75 × 50–175 × 100 × 100 μm (i) ca. 100 × 50 × 50 μm (ii) and (iii) 100 × 75 × ?–180 × 125 × ? μm	

Question marks refer to dimensions that could not be accurately determined due to alignment of a crystal axis with the vertical direction.

**Figure A1:**
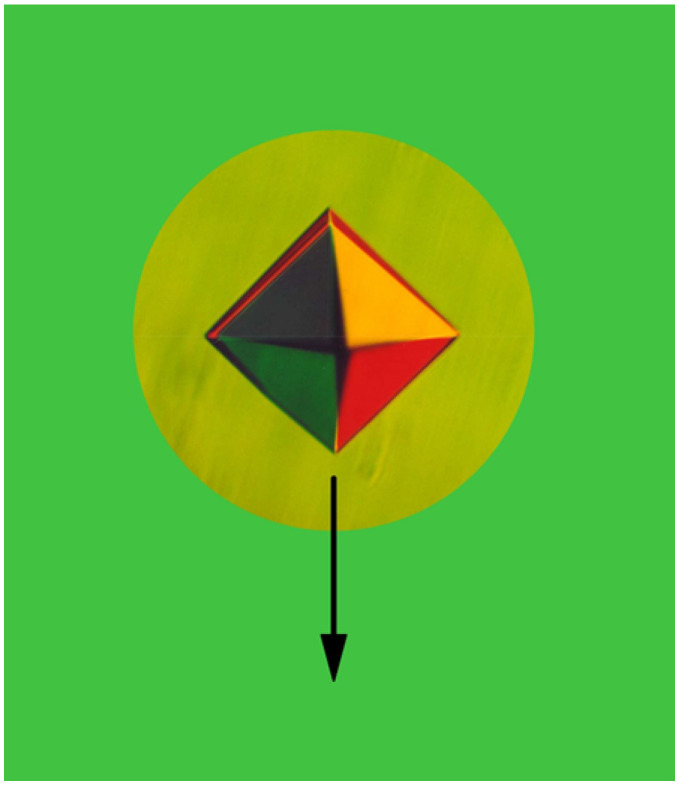
A crystal, born in the solution bulk, sediments (shown by the arrow); thus, the crystal leaves the depleted solution zone (yellow) formed around it. As a result, the crystal starts growing faster in the nondepleted solution (green). However, when they reach the bottom of the container, the crystals can land either in nondepleted or in already-depleted solution. Microphotograph of a real (interference contrasted) crystal of apoferritin (edge length 0.25 mm) is used for showing the settling crystal. Reprinted with permission from [17].

## Data Availability

All supporting data are reported in the text.

## Appendix A. Crystal Size Distribution

Although the size difference in the nascent crystals can be significant at the nanoscale, if preserved at the same level during the crystal growth itself, this difference would fade as we move into the macroscale. Consequently, all grown crystals would be indistinguishable in size, which is not the case [17]. Evidently, the polydispersity introduced during the nucleation stage increases during the subsequent crystal growth, which makes the CSD increasingly nonuniform. This happens for many reasons, which, however, can be divided into two categories: controllable (such as the amount of dissolved substance, the solubility, and the growth time) and noncontrollable factors, which follow:

− The competition for material (needed for growth) between crystals that are positioned close to each other. There are indications [33], however, that presumably due to the relatively slow protein crystal growth, the competition for solute is not very intense and this alone can hardly be the major cause for protein crystal polydispersity and its gradual increase during prolonged growth.
− The plumes [23,25] that arise because solution with lower solute concentration around the growing crystal rises, and fresh, more concentrated solution from farther away invades that space; the larger the growing crystal, the larger the plume forming above it, in other words the more extensive the solution replenishment around the crystal.
− Step sources of increased growth activity (such as closely spaced screw dislocations of the same and opposite signs) are present in some large crystals while absent in others [33]. Such defects should be absent in nanocrystals, and this has been explained by estimating the equilibrium distance between two dislocations [34]; with crystal size decreasing below the equilibrium separation distance, dislocations inside such nanocrystals become unstable.
− The crystals that are born first in the solution bulk sediment (Figure A1), which brings them into the non-depleted solution where they grow faster. Crystal sedimentation occurs when the viscous resistance cannot counterbalance the gravitational drag force acting on the protein crystal. Therefore, when crystals grow to sizes between 1.6 μm [35] and 2 to 6 μm [36], most of them settle to the bottom and continue to grow there. However, sedimentation also destroys the Chernov ”self-purifying” zones and exposes the growing crystals to increased impurity delivery.
